# Correction to “Health Equity in Systematic Reviews: A Tutorial—Part 1 Getting Started With Health Equity in Your Review”, “Health Equity in Systematic Reviews: A Tutorial—Part 2 Implementing Health Equity Throughout Your Methods”, “Meta‐Analysis Using Time‐to‐Event Data: A Tutorial” and “Split Body Trials in Systematic Reviews and Meta‐Analyses: A Tutorial”

**DOI:** 10.1002/cesm.70062

**Published:** 2026-02-19

J. Petkovic, J. P. Pardo, V. Welch, et al., “Health Equity in Systematic Reviews: A Tutorial—Part 1 Getting Started With Health Equity in Your Review,” *Cochrane Evidence Synthesis and Methods* 3 (2025): 1–6, https://doi.org/10.1002/cesm.70055.

J. Petkovic, J. P. Pardo, V. Welch, et al., “Health Equity in Systematic Reviews: A Tutorial—Part 2 Implementing Health Equity Throughout Your Methods,” *Cochrane Evidence Synthesis and Methods* 3 (2025): 1–7, https://doi.org/10.1002/cesm.70054.

Krishan, A. and Dwan, K. (2025), Meta‐Analysis Using Time‐to‐Event Data: A Tutorial. Cochrane Evidence Synthesis and Methods, 3: e70041. https://doi.org/10.1002/cesm.70041

Livingstone, N., Dwan, K. and Chaplin, M. (2025), Split Body Trials in Systematic Reviews and Meta‐Analyses: A Tutorial. Cochrane Evidence Synthesis and Methods, 3: e70052. https://doi.org/10.1002/cesm.70052

The two articles by Petkovic *et al.* (2025) were incorrectly labelled as “Methods Article”. They have now been corrected to “Tutorial”.

In Petkovic *et al.* (2025), Appendix A, the row for ‘Religion’ states “This refers to the religious beliefs and can affect health equity when the choices related to these beliefs are imposed on a person by their family or community”. This should state “This refers to religious beliefs, which can influence health and well‐being by fostering community support and shared values, and may also affect health equity (positively and negatively) when choices related to these beliefs are shaped by family or community expectations”.

In Petkovic *et al.* (2025), the following Conflict of Interest statement was missing:

**CONFLICT OF INTEREST STATEMENT**

All authors are members of the leadership team of the Cochrane Health Equity Thematic Group. The authors have no other conflicts to declare.

The article by Krishan & Dwan (2025) was incorrectly labelled as “Brief Report”. This has now been corrected to “Tutorial”.

The article by Livingstone, Dwan & Chaplin (2025) was incorrectly labelled as “Editorial”. This has now been corrected to “Tutorial”.

These have now been corrected in the published articles.

We apologize for these errors.
